# ‘Test n Treat (TnT)’: a cluster-randomised feasibility trial of frequent, rapid-testing and same-day, on-site treatment to reduce rates of chlamydia in high-risk further education college students: statistical analysis plan

**DOI:** 10.1186/s13063-018-2675-7

**Published:** 2018-06-05

**Authors:** Rachel Phillips, Pippa Oakeshott, Sarah Kerry-Barnard, Fiona Reid

**Affiliations:** 1 School of Population Health & Environmental Sciences, Faculty of Life Sciences and Medicine, King’s College London, London, UK; 2 Population Health Research Institute St George’s, University of London, London, UK

**Keywords:** Statistical analysis plan, Feasibility study, Randomised controlled trial, Rapid chlamydia tests, Screening, Young people, Further education colleges, Test n treat

## Abstract

**Background:**

There are high rates of sexually transmitted infections (STIs) in ethnically diverse, sexually active students aged 16–24 years attending London further education (FE) colleges. However, uptake of chlamydia screening remains low. The TnT study aims to assess the feasibility of conducting a future trial in FE colleges to investigate if frequent, rapid, on-site testing and treatment (TnT) reduces chlamydia rates. This article presents the statistical analysis plan for the main study publication as approved and signed off by the Trial Management Group prior to the first data extraction for the final report.

**Methods/design:**

TnT is a cluster-randomised feasibility trial conducted over 7 months with parallel qualitative and economic assessments. Colleges will be randomly allocated into the intervention (TnT) or the control group (no TnT). Six FE colleges in London will be included. At each college for 2 days, 80 consecutive sexually active students aged 16–24 years (total 480 students across all six colleges) will be recruited from public areas and asked to provide baseline samples. One and 4 months after recruitment intervention colleges will be visited on two consecutive days by the TnT team where participating students will be texted and invited to come for same-day, on-site, rapid chlamydia testing and, if positive, treatment. Participants in the control colleges will receive ‘thank you’ texts 1 and 4 months after recruitment. Seven months after recruitment, participants from both groups will be invited to complete questionnaires and provide samples for TnT. All samples will be tested, and same-day treatment offered to participants with positive results. Key feasibility outcomes include: recruitment rates, testing and treatment uptake rates (at 1 and 4 months) and follow-up rates (at 7 months).

**Trial registration:**

ISRCTN 58038795. Registered on 31 August 2016.

## Background

There are high rates of sexually transmitted infections (STIs) in ethnically diverse, sexually active students aged 16–24 years attending London further education (FE) colleges [1–4], with around 8% testing positive for *Chlamydia trachomatis*. However, uptake of chlamydia screening remains low: below 30% annually in 16–24-year-olds in England [4, 5]. Although chlamydia and gonorrhoea primarily affect young people, the consequences of infection such as infertility, chronic pelvic pain or epididymitis can last a lifetime. It is estimated that 10–16% of women with untreated chlamydia will develop clinical pelvic inflammatory disease of whom 8% will have an ectopic pregnancy and 11% will suffer from tubal-factor infertility [6]. The cost of chlamydia and gonorrhoea to the NHS is estimated to be over £100 million each year.

Barriers to reducing chlamydia rates include low uptake of testing by those most at risk [5, 7] (such as teenagers, people from ethnic minorities and people who are socioeconomically deprived), and long delays in receiving a positive diagnosis or attending for treatment [8, 9]. Introducing rapid, on-the-spot chlamydia tests and treatment into the community could make it easier for young people to get tested and treated faster before they can pass on their infection. It might also prevent complications [10, 11]. These novel tests can have 99% sensitivity and 99.4% specificity [12], and studies have demonstrated their feasibility in remote communities [13]. However, there have been no UK trials of rapid sexually transmitted infection (STI) tests and same-day, on-site treatment in non-healthcare settings.

## Objective

To assess the feasibility of conducting a future trial in further education (FE) colleges to investigate if frequent, rapid, on-site testing and treatment (TnT) reduces chlamydia rates in sexually active male and female students aged 16–24 years.

## Methods and design

TnT is a cluster-randomised feasibility trial conducted over 7 months with parallel qualitative and economic assessments. The outcome is measured within one academic year to optimise follow-up.

Both the current feasibility study and a future, definitive trial will be cluster randomised. This is to avoid contamination, which may arise if students were individually randomised within the same college, as sexual partners could potentially be allocated to different groups. The cluster design also reflects how TnT would be rolled out in practice, with college visits once each term.

Six FE colleges in London will be included. Colleges were included based on their high proportion of black and ethnic minority students and an even gender split, as well as their close proximity to St George’s, University of London (SGUL) [3]. At each college, for 2 days, 80 consecutive sexually active students aged 16–24 years will be recruited from public areas (total 480 students across all six colleges). Research assistants will approach students in common room areas. The students will be asked if they are willing to help with research on sexual health. Potentially eligible students will be invited to come to the study table where recruiters will explain that as the study is about chlamydia and sexually transmitted infections, only students who have had penetrative sexual intercourse should consider taking part. Those who are interested will be given a patient information sheet and consent form to read and encouraged to ask questions.

### Exclusion criteria

- Students who self-report never having had penetrative sexual intercourse
- Students with severe learning disability as identified by college identification badges

Randomisation will take place once recruitment is completed and baseline data collected for all colleges. Colleges will be randomly allocated into the intervention (TnT) or the control group (no TnT) in a 1:1 (i.e. equal allocation) ratio by the trial statistician. The randomisation will be constrained to ensure that three colleges are allocated to each group. Recruitment of colleges and participants will take place prior to group allocation to ensure allocation concealment and prevent selection bias, and, therefore, the baseline data collection from students will be blind to treatment group.

Participants will be asked to provide samples (urine for males and self-taken vaginal swabs for females) at baseline and after 7 months and to complete questionnaires on sexual lifestyle and healthcare use at both time points. Questionnaire data will be collected at the college using encrypted tablet computers. As a contingency, paper questionnaires will be used as back up. All participants will be informed that baseline samples will not be tested for 7 months and will be advised to get screened separately from the study in the event that they are allocated to the control group.

One month after recruitment, each intervention campus will be visited on two consecutive days by the TnT team. (These will be the same days of the week as at recruitment to optimise participating student attendance.) The 80 participating students in each campus will be invited to provide a sample, but this time the sample will be tested immediately on site using the Cepheid GeneXpert system which takes 90 min. Participants will be given a card designed by the user group containing information about STIs, and links to the Brook sexual health website: www.brook.org.uk. Negative results will be sent to participants by text. Participants with positive chlamydia results will be telephoned and invited to come to the college nurse’s room for treatment, partner notification, advice and follow-up by a visiting dispensing (under Patient Group Directive) nurse health adviser. Participants with infections will be asked to bring any sexual partners who attend the college so they can also be tested and treated. The nurse health adviser will arrange for participants who are positive for gonorrhoea to be reviewed by a clinician on the same or the next day at a genitourinary medicine (GUM) clinic. This is for clinical examination, so that repeat samples can be taken to evaluate gonorrhoea resistance to antibiotics, for intramuscular injection of antibiotics and for partner notification. All participants in the three intervention campuses will be invited to provide repeat samples for on-site TnT 4 months after recruitment (i.e. the next college term), when all the procedures described above will be repeated.

Participants from the three control colleges will not get TnT but will receive texts 1 and 4 months after recruitment thanking them for being in the study.

All participants will be asked to provide samples and to complete questionnaires at college at 7 months. Testing at 7 months is required in the proposed full trial to calculate the main outcome (prevalence of chlamydia), and is included here to test the feasibility of collecting these data. Treatment will also be offered to those diagnosed with infection at 7 months. This is not part of the assessed intervention, but is offered to enhance screening uptake, and participation in general among the control group. In addition, at the end of the study, stored baseline samples will be tested using standard tests, and participants with positive results will be contacted by the health adviser. Those not attending for follow-up will be sent an SMS text with a link to the final questionnaire to be completed online. They will also be telephoned and offered the opportunity to provide a sample for routine (not rapid) testing either in college at a prearranged time or by post.

## Outcomes

1. The outcomes of this study include key values to inform feasibility, sample size and timescales of a full trial of TnT in FE colleges. These include:
  - Recruitment rates and associated outcomes:
    Of the total number of students assessed for eligibility the proportion who are eligible are asked to participate in the study
    Of those eligible the proportion recruited to the study
    The time to recruit 80 students at each college
    Age, gender and ethnicity of students recruited versus students not recruited
  - Testing and Treatment uptake rates (1 and 4 months after recruitment) (intervention colleges only):
    Of the total number of students recruited in the intervention group the proportion that return at 1 month (and 4 months) and provide a sample for testing
    Of those tested, the proportion with positive test results and the proportion treated
    The time from test to informing the participating student of the result
    The time from test to treatment of positives
    The number of partners confirmed treated per index case
  - Follow-up rates (at 7 months):
    Of the total number of students recruited the proportion that return at month 7 and provide a sample for testing
    Of those tested, the proportion with positive test results and the proportion treated
    Of the total number of students recruited, the proportion that complete the final questionnaires (including data on healthcare usage)
  - Prevalence of chlamydia in participants at each college at baseline and at 7 months
2. A perspective on the acceptability of TnT in FE colleges emerging from qualitative interviews, including barriers and facilitators to uptake and possible harms. This will be described in more detail elsewhere.
3. Estimate of the cost per person screened and treated in TnT versus usual care. Detailed analysis plans for these health economic assessments will be documented separately by the trial’s health economist.

## Sample size calculation

Assuming a 30% recruitment rate [14], 1600 students will be approached to recruit 480 overall (80 per college across six colleges: three intervention colleges and three control colleges). Estimates of testing uptake at 1 and 4 months (intervention colleges only) will be based on 240 students, and at 7 months will be based on 480 students (all colleges).

Teare et al. recommend that 60 to 100 subjects are sufficient to estimate an event rate with acceptable precision in a feasibility study [15]. Prevalence of chlamydia at baseline will be estimated separately for each of the six colleges (80 students per college), and these prevalence figures will be used to inform the intraclass correlation coefficient (ICC), required for the sample size calculation for the main study. From our previous research involving 11 colleges, the ICC was estimated to be 0.005 (95% confidence interval − 0.013 to 0.026) [3]. Adding data from another six colleges will improve the precision of this ICC, reducing the width of the confidence interval by around 20%.

Assuming 70% followed up at 7 months, final estimates of chlamydia prevalence would be based on 168 students in each of the intervention and control groups [3]. The study is not powered to find a statistically significant difference in chlamydia rates between groups, but may provide useful information on possible effect size to inform future sample size calculations.

## Statistical analyses

### Trial profile

**Fig. 1:**
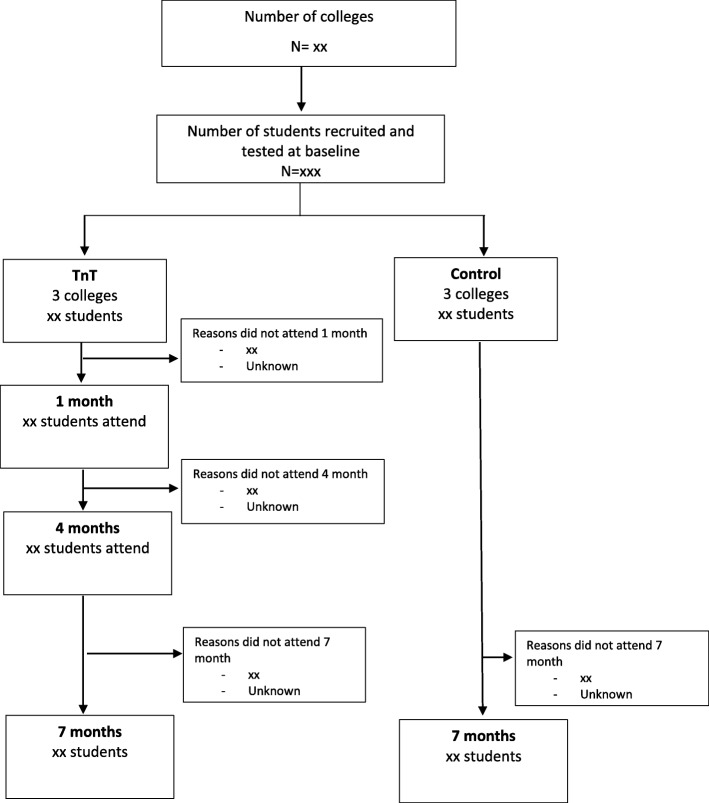
Consolidated Standards of Reporting Trials (CONSORT) trial flowchart

The flow of participants will be displayed in the Consolidated Standards of Reporting Trials (CONSORT) flowchart as shown in Fig. 1 [16].

### Data management and quality assurance

Contact details will be collected on paper consent forms which will then be entered and stored on an encrypted Access database by authorised research personnel. Attendance and laboratory data will be initially recorded on paper worksheets and then entered, by designated research personnel, to an online database (REDCap) [17] hosted by SGUL. Questionnaire data will be entered electronically using the REDCap mobile application on encrypted tablets where tablets are available, or on paper as a back-up. At the end of the visit to each college, the data will be uploaded or entered to the SGUL servers by designated research personnel.

Research coordinators and the trial manager will periodically perform basic checks, such as examining for improbable values and data completeness, as well as random checks on the data. Anomalies will be explored and checked with original source data.

After the last patient has been followed up, all queries resolved and all data fields completed with data or missing data codes, the database will be locked for final analysis and this process will be overseen by the trial statisticians.

### General analysis principles

The analysis principles outlined will be followed as closely as possible in the analysis and reporting of trial data; the statistical analysis plan is not intended to restrict exploratory or other sensible and standard reporting practices. There are no plans for any formal comparisons between treatment groups until final database lock. Analysis will be undertaken by the trial statistician.

Since this a feasibility study no statistical significance testing will be performed. Any confidence intervals presented will be two-sided at the 95% confidence level.

### Baseline characteristics

Baseline descriptions of students recruited and tested will be presented by treatment group: including means and standard deviation or numbers and proportions as appropriate. This will include demographic characteristics, history of sexual behaviours and chlamydia and gonorrhoea status (a full list is available in Appendix). These summaries will be based on observed values and the number of missing observations for each characteristic will be reported.

### Main outcomes

#### Recruitment rates (at baseline)

1. We will assess eligibility rates by calculating the proportion of students who were eligible and were asked to participate in the study out of the total number of students who were assessed for eligibility. Similarly, the proportion of students who were recruited to the study out of those eligible will be calculated. All proportions will be presented with corresponding 95% confidence intervals (CIs).
2. The time taken to recruit 80 participants at each college will be described. We will calculate the mean and standard deviation or medians and inter-quartile ranges as appropriate of the time taken to recruit 80 participants at each college.
3. Age, gender and ethnicity at baseline will be presented separately for those recruited to the study and those eligible but not recruited. We will present means and standard deviation or numbers and proportions as appropriate.

#### Testing and treatment uptake rates, in intervention colleges only (1 and 4 months after recruitment)

1. The number and proportion of participants in the intervention group who return at 1 month and provide a sample out of the total recruited to the intervention group will be presented. Of those tested, the number and proportion of participants with positive test results and the number and proportion of participants treated will be presented. These analyses will be repeated for the 4-month visit. Corresponding 95% CIs will be presented. Where available, the reasons for non-attendance will be summarised
2. Time from test to treatment of positives will be described. Of the participants who attended the 1-month intervention visit, were tested and received a positive test result we will calculate the mean and standard deviation or medians and inter-quartile ranges as appropriate of the time between the sample being taken and the time treatment was received. This will be repeated for the 4-month visit.
3. The number of partners confirmed treated per index case will be described. In addition, we will report the number of partner notifications raised and addressed, the number of participants who confirm partner treated, the number of participants referred to a GUM clinic and, therefore, partner notification responsibility passed to clinic and the number of participants with no information regarding treatment.

#### Follow-up rates (at 7 months)

1. The number and proportion of participants who return at month 7 and provide a sample out of the total recruited will be presented by treatment group. Of those tested, the number and proportion of participants with positive test results and the number and proportion of participants treated will be presented. Corresponding 95% CIs will be presented.
2. The number and proportion of participants completing the final questionnaire will be presented. Descriptions of participants’ responses by treatment group will be presented (including data on healthcare usage), by means and standard deviation or numbers and proportions as appropriate. We will present the number of repeat follow-up attempts for non-attendees at 7-month follow-up.

#### Prevalence of chlamydia in participants at each college at baseline and at 7 months

Of those tested, the number and proportion of participants with positive test results will be presented for each college at baseline and at 7 months.

#### Sensitivity analysis

##### Missing data analysis

As this is a feasibility study the levels of data completion and follow-up rates are important feasibility outcomes. Therefore, no formal analysis will be undertaken to account for missing data. All summaries will be based on observations only and the number of missing observations for each characteristic will be reported.

### Harm data

Participating student and college staff views of potential harms of on-site rapid tests and treatment will be sought and examined in the qualitative interviews. Analysis details will be described elsewhere.

## Trial status

Recruitment was completed in October 2016. Final follow-up concluded in August 2017.

## Conclusion

Findings from this study are intended to assess the feasibility of running a future definitive trial to investigate whether a FE college-based TnT model leads to a reduction in prevalence of chlamydia. In this article, we have described the TnT statistical analysis plan which provides details about how data from the TnT feasibility trial will be analysed. The protocol for the trial is published by Kerry-Barnard et al. (in press). By publishing our statistical analysis plan, we believe that we will ensure a more balanced, accurate and complete report of our final results.

## Appendix

### Baseline characteristics

The following baseline characteristics will be summarised by treatment group:

1. Age
2. Sex
3. Ethnicity
4. Female sexual preference
5. Male sexual preference
6. Age at first sex
7. Number of sexual partners in last year
8. New sexual partner in the past 6 months
9. Type of female contraception
10. Condom use
11. Last sexually transmitted infection (STI) check-up
12. STI history
13. Antibiotics received for an infection in the last 2 weeks
14. Which antibiotics
15. Any symptoms in the past 6 months (female)
16. Any symptoms in the past 6 months (male)
17. Smoker status
18. Vape
19. Alcohol consumption in past month
20. Visited GP in the past 6 months
21. Number of GP visits in the past 6 months
22. Reason for GP visit
23. Visited GUM clinic in the past 6 months
24. Number of GUM clinical visits in the past 6 months
25. Visited walk-in clinic in the past 6 months
26. Number of visits to a walk-in clinical in the past 6 months
27. Visited A&E/hospital in the past 6 months
28. Number of visits to A&E/hospital in the past 6 months
29. Attended healthcare facility for sexual health reasons

## Abbreviations

CI: Confidence interval
CONSORT: Consolidated Standards of Reporting Trials
FE: Further education
GUM: Genitourinary medicine
ICC: Intraclass correlation coefficient
REDCap: Research Electronic Data Capture
SGUL: St George’s, University of London
STI: Sexually transmitted infections
TnT: Test n Treat
